# Supplementary material for: Spatial localization of hippocampal replay requires dopamine signaling
Source: bioRxiv. 2024 Nov 8:2024.06.04.597435. Originally published 2024 Jun 6. Preprint. [Version 2] doi: 10.1101/2024.06.04.597435 (PMC11185723; doi:10.1101/2024.06.04.597435)
Supplement: 1 [file NIHPP2024.06.04.597435V2-supplement-1.pdf]

bioRxiv preprint doi: https://doi.org/10.1101/2024.06.04.597435; this version posted November 8, 2024. The copyright holder for this preprint (which was not certified by peer review) is the author/funder, who has granted bioRxiv a license to display the preprint in perpetuity. It is made available under aCC-BY 4.0 International license.

## Supplemental Figure Legends

**Figure 1 – Supplement 1. Additional histology examples.**
**(A)** Additional example section from experimental rat with evident virus expression. Scale bar 300 µm. Dashed line marks approximate location of midline. mCherry puncta (top panel) and TH-positive dopaminergic neurons (bottom panel) indicated virus expression in the VTA. **(B)** Additional example section from control rat with evident virus expression. Scale bar 150 µm. **(C)** Example sections from 1st rat excluded due to lack of virus expression. Scale bar 600 µm. Complete lack of mCherry puncta (top panel) indicated failure of virus expression. **(D)** Example sections from 2nd rat excluded due to lack of virus expression. Scale bar 300 µm.

**Figure 1 – Supplement 2. Behavioral effects of novelty and VTA inactivation.**
**(A)** In novel sessions, Unch. visit duration decreased from Epoch 1 to Epoch 2, while CNO additionally led to longer visit duration in experimental rats. Mean ± standard error, Exp Saline, Epoch 1: 7.551±1.11, Epoch 2: 4.98±0.44; Exp CNO, Epoch 1: 12.14±1.7, Epoch 2: 6.75±0.64. Con Saline, Epoch 1: 7.76±0.65, Epoch 2: 4.86±0.35; Con CNO, Epoch 1: 7.46±0.59, Epoch 2: 4.96±0.25. Mixed-effects GLM with epoch, drug, animal group, and all interactions, with individual animal as a random effect: epoch, $z=-2.99$, $p<0.01$; group X drug, $z=3.29$, $p<0.01$; all other terms, n.s. **(B)** In novel sessions, Incr. visit duration increased from Epoch 1 to Epoch 2, while CNO additionally led to longer visit duration in experimental rats. Mean ± standard error, Exp Saline, Epoch 1: 7.52±1.32, Epoch 2: 10.05±0.88; Exp CNO, Epoch 1: 11.55±2, Epoch 2: 13.39±1.18. Con Saline, Epoch 1: 7.77±0.58, Epoch 2: 10.97±0.55; Con CNO, Epoch 1: 6.77±0.38, Epoch 2: 10.46±0.22. Mixed-effects GLM with epoch, drug, animal group, and all interactions, with individual animal as a random effect: epoch, $z=2.82$, $p<0.01$; group X drug, $z=2.91$, $p<0.01$; all other terms, n.s. **(C)** In familiar sessions, Unch. visit duration decreased from Epoch 1 to Epoch 2, with only a modest effect of CNO compared to novel sessions. Mean ± standard error, Exp Saline, Epoch 1: 6.45±0.72, Epoch 2: 5.17±0.66; Exp CNO, Epoch 1: 6.47±0.42, Epoch 2: 5.841±0.51. Con Saline, Epoch 1: 6.54±0.5, Epoch 2: 4.72±0.37; Con CNO, Epoch 1: 6.18±0. 3, Epoch 2: 4.17±0.17. Mixed-effects GLM with epoch, drug, animal group, and all interactions, with individual animal as a random effect: epoch, $z=-2.37$, $p<0.05$; all other terms, n.s. **(D)** In familiar sessions, Incr. visit duration increased from Epoch 1 to Epoch 2. Mean ± standard error, Exp Saline, Epoch 1: 6.41±0.59, Epoch 2: 11.1±0.97; Exp CNO, Epoch 1: 6.03±0.18, Epoch 2: 11.99±1. Con Saline, Epoch 1: 7.27±0.66, Epoch 2: 11.46±0.71; Con CNO, Epoch 1: 6.33±0.48, Epoch 2: 10.28±0.4. Mixed-effects GLM with epoch, drug, animal group, and all interactions, with individual animal as a random effect: epoch, $z=3.97$, $p<0.001$; all other terms, n.s. **(E)** Unch. visit duration increased from Epoch 2 to Epoch 3. Mean ± standard error, Exp Saline, Epoch 2: 5.13±0.53, Epoch 3: 7.95±0.6; Exp CNO, Epoch 2: 6.04±0.42, Epoch 3: 7.89±0.68. Con Saline, Epoch 2: 4.77±0.27, Epoch 3: 6.23±0.37; Con CNO, Epoch 2: 4.43±0.15, Epoch 3: 5.96±0.19. Mixed-effects GLM with epoch, drug, animal group, and all interactions, with individual animal as a random effect: epoch, $z=2.32$, $p<0.05$; all other terms, n.s. **(F)** Incr. visit duration decreased from Epoch 2 to Epoch 3. Mean ± standard error, Exp Saline, Epoch 2: 10.87±0.8, Epoch 3: 9.08±1.01; Exp CNO, Epoch 2: 12.31±0.82, Epoch 3: 9.92±0.95. Con Saline, Epoch 2:

bioRxiv preprint doi: https://doi.org/10.1101/2024.06.04.597435; this version posted November 8, 2024. The copyright holder for this preprint (which was not certified by peer review) is the author/funder, who has granted bioRxiv a license to display the preprint in perpetuity. It is made available under aCC-BY 4.0 International license.

11.29±0.5, Epoch 3: 6.48±0.28; Con CNO, Epoch 2: 10.34±0.27, Epoch 3: 6.42±0.29. Mixed-effects GLM with epoch, drug, animal group, and all interactions, with individual animal as a random effect: epoch, $z=-4.77$, $p<10^{-5}$, group X epoch, $z=2.31$, $p<0.05$; all other terms, n.s.

**Figure 1 – Supplement 3. Effect of reward change on running velocity.** Running speed towards the Incr. end in Epoch 2 was consistently significantly faster than towards the Unch. end, across all conditions. The median running speed in all non-zero velocity timepoints while the animal was located outside of the reward end zones in each epoch and running direction was calculated for each session. Mean and standard error across sessions are shown here. Mixed-effects model predicting the velocity difference in Epoch 2, Incr. – Unch., as a function of drug, novelty, and their interaction, with animal-specific intercepts. Experimental group : intercept, $z=2.99$, $p<0.01$; drug, $z=3.18$ , $p<0.01$; all other terms, n.s. Control group: intercept, $z=7.85$, $p<10^{-10}$; all other terms, n.s. Filled symbol, saline; unfilled symbol, CNO.

**Figure 2 – Supplement 1. Modulation of SWR rate by reward increase.** **(A)** In experimental rats, a mixed effects Poisson GLM was fit to the data and 5,000 drug identity shuffles. The difference between model-predicted SWR rate in saline and CNO sessions at each reward end (Unch. top row, Incr. bottom row) and novelty condition (familiar left column, novel right column), in data (red lines) and in bootstrap shuffles (histogram). Significance values reflect one-tailed hypothesis test, with hypotheses that Unch. saline < Unch. CNO and Incr. saline > Incr. CNO. **(B)** A mixed effects GLM with bootstrap, as in (A), but for control animals.

**Figure 2 – Supplement 2. SWR rate in Epoch 3.** **(A)** SWR rate as a function of time in stopping period in Epoch 2 and 3 for four example sessions in experimental rats, as in Figure 2a. Epoch 2 (red lines), Epoch 3 (dashed gray lines). SWR rate was binned in 0.25 s windows and smoothed with a 2 bin Gaussian. Line, mean; shading, standard error. **(B)** Same as Figure 2F, but for Epoch 3. **(C)** Same as Figure 2G, but for Epoch 3.

**Figure 2 – Supplement 3. Similar results independent of session number of the day.** The dataset for Experiment 1 was split into two, with one part including only sessions that occurred 1st in any given day (“1st of day”) and the other including all sessions that were not the 1st in any given day (“2nd+ of day”). Although the low resultant session counts in each group precluded statistical analysis, the main effect of CNO in reducing SWR rate difference between the reward ends was very similar.

**Figure 2 – Supplement 4. Ripple duration is increased with familiarity.** The duration of SWR was examined similarly to the analysis on SWR rate. A mixed-effects Poisson generalized linear model (GLM) was fit to predict changes in SWR duration across reward end, epoch, drug condition, and novelty, with animal identity as a random effect. Significant coefficients: CNO ($z=-5.86$, $p<10^{-5}$) and Epoch 2 ($z=-2.1$, $p<0.05$).

**Figure 3 – Supplement 1. SWR rate at stable end in experimental rats.**

bioRxiv preprint doi: https://doi.org/10.1101/2024.06.04.597435; this version posted November 8, 2024. The copyright holder for this preprint (which was not certified by peer review) is the author/funder, who has granted bioRxiv a license to display the preprint in perpetuity. It is made available under aCC-BY 4.0 International license.

**(A)** At stable end visits in saline sessions, SWR rate was not significantly modulated by the previous volatile end visit reward volume. Pearson correlation between SWR rate and previous volatile volume, r=-0.0643, p=0.21. Two sample t-test between volatile volume ≤ 2 and volatile volume > 2, t(380)=1.465, p=0.144. **(B)** At stable end visits in CNO sessions, SWR rate was not significantly modulated by the previous volatile end visit reward volume. Pearson correlation between SWR rate and previous volatile volume, r=-0.0645, p=0.205. Two sample t-test between volatile volume ≤ 2 and volatile volume > 2, t(386)=1.137, p = 0.256. Two-way ANOVA with drug and previous volatile volume ≤ 2: drug (F[1,766]=6.43, p<0.05), volume ≤ 2 (F[1,766]=3.36, p=0.067), drug X volume (F[1,766]=0.03, p=0.853). Error bars, standard error.

**Figure 3 – Supplement 2. SWR rate in all sessions in volatile reward task.**
**(A)** SWR rate as a function of reward volume and time in end visit, as in Figure 3B, for all sessions combined (including saline and CNO sessions in experimental and control rats). Left panel, stable reward end. Right panel, volatile reward end. In stable panel, traces are colored based on previous volatile end visit volume. In volatile panel, traces are colored based on current volatile volume. **(B)** SWR rate at volatile end as a function of current and previous volatile volume, as in Figure 3D, for all volatile reward task sessions. **(C)** SWR rate for each non-zero volatile volume plotted as a function of previous volume, with the mean SWR rate for that current volume subtracted. Unfilled symbols, mean of previous volume across all current volumes. Thick dashed line, linear fit to mean values. Pearson correlation between (ripple rate – mean) and previous volume, r=-0.07, p<0.01, consistent with RPE coding. Error bars, standard error. **(D)** Positive RPE caused significantly greater ripple rate than negative RPE (two-sample t-test, t[1661]=2.741, p<0.01). **(E)** SWR rate at the stable end was significantly negatively correlated with the most recent volatile volume (r=-0.06, p<0.01). **(F)** SWR rate at the stable end was significantly greater when the most recent volatile end volume was less than or equal in volume (≤ 2) than when it was greater (two-sample t-test, t[2485]=2.582, p<0.01). **(G)** SWR rate at the volatile end was significantly higher if recent reward history was lower than the average. Reward volume at the 3 previous visits was averaged, then split above and below the median. Poisson GLM with two terms, current volume and reward history (above/below median): current volume, z=22.21, $p<10^{-10}$; history, z=-2.03, p<0.05).

**Figure 4 – Supplement 1. Effect of novelty and VTA inactivation on place cell properties.**
**(A)** Correlation between single lap place fields and session averaged field. Three-way ANOVA with drug, novelty, and animal group: novelty (F[1,3249]=6.75, p<0.01), novelty X group (F[1,3249]=15.76, p<0.01), all others, p>0.2. **(B)** Correlation between unidirectional fields calculated separately in each running direction. Three-way ANOVA with drug, novelty, and animal group: drug (F[1,2816]=5.76, p<0.05), novelty (F[1,2816]=28.21, $p<10^{-10}$), drug X novelty (F[1,2816]=5.52, p<0.05), novelty X group (F[1,2816]=6.56, p<0.05), all others, p>0.17.

**Figure 4 – Supplement 2. Run decoding accuracy in replay analysis sessions.**

bioRxiv preprint doi: https://doi.org/10.1101/2024.06.04.597435; this version posted November 8, 2024. The copyright holder for this preprint (which was not certified by peer review) is the author/funder, who has granted bioRxiv a license to display the preprint in perpetuity. It is made available under aCC-BY 4.0 International license.

**(A)** Mean decoding error during run. Position and running direction were decoded during periods of strong locomotion (animal velocity >20 cm/s and position >20 cm from the reward wells) in 250 ms bins. Sessions with >35 cm mean decoding error were excluded from analysis. A mixed-effects model predicting position decoding error as a function of drug, novelty, recorded neuron count, and mean place field size, found more recorded cells and smaller mean field size in both groups led to smaller decoding errors. Experimental rats: drug, z=-2.48, $p<0.05$; cell count, z=-3.48, $p<0.01$; mean field size, z=5.36, $p<10^{-5}$; other terms, n.s. Control rats: cell count, z=-4.07, $p<0.01$; mean field size, z=4.04, $p<0.01$; other terms, n.s. Filled and unfilled symbols are saline and CNO sessions, respectively. Error bars, standard error. **(B)** Mean fraction of bins where actual and decoded running direction were the same. Sessions with <60% match were excluded from analysis. Symbols as in (A). A mixed-effects model predicting probability of matching real and decoded run direction as a function of drug, novelty, recorded neuron count, and mean place field size, found more recorded cells in both groups led to higher match probability. Experimental rats: drug, z=2.33, $p<0.05$; novelty, z=-2.96, $p<0.01$; cell count, z=6.81, $p<10^{-5}$; other terms, n.s. Control rats: cell count, z=6.23, $p<10^{-5}$; other terms, n.s.

**Figure 4 – Supplement 3. Non-local replay was unaffected by experimental manipulations.**

**(A)** The difference in rate of reverse replay at each end (Incr. – Unch.) in novel sessions in experimental rats. Error bars, standard error of the mean. Reward condition is indicated by color (equal reward, epoch 1 and 3, gray; unequal reward, epoch 2, orange), and drug condition is indicated on the x-axis. The difference in replay rate between equal and unequal reward conditions was assessed with a mixed-effects linear model with drug, novelty, and replay directionality, and animal-specific intercepts as random effect: all terms, n.s. **(B)** Same as (A), but for familiar sessions. **(C)** Same as (A), but for forward replay. Unequal reward, epoch 2, purple. **(D)** Same as (C), but for familiar sessions.

bioRxiv preprint doi: https://doi.org/10.1101/2024.06.04.597435; this version posted November 8, 2024. The copyright holder for this preprint (which was not certified by peer review) is the author/funder, who has granted bioRxiv a license to display the preprint in perpetuity. It is made available under aCC-BY 4.0 International license.

## Supplemental Figures

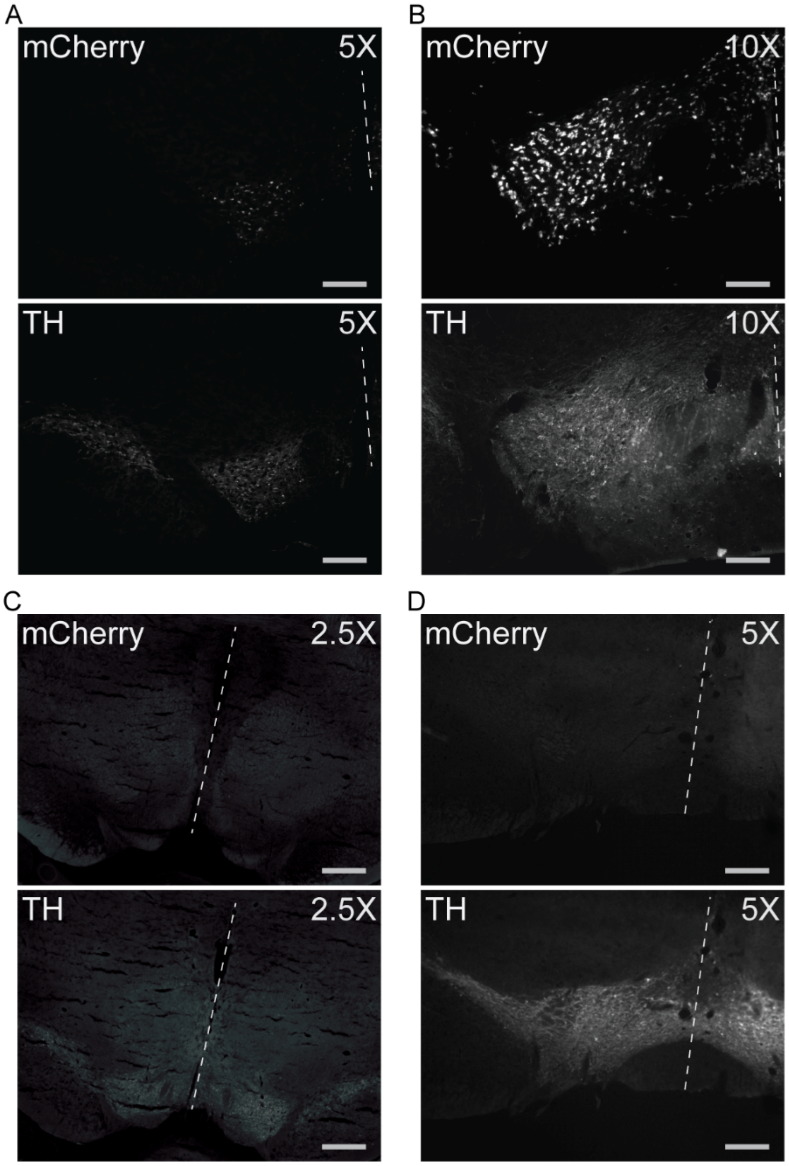


**Figure 1 – Supplement 1**

bioRxiv preprint doi: https://doi.org/10.1101/2024.06.04.597435; this version posted November 8, 2024. The copyright holder for this preprint (which was not certified by peer review) is the author/funder, who has granted bioRxiv a license to display the preprint in perpetuity. It is made available under aCC-BY 4.0 International license.

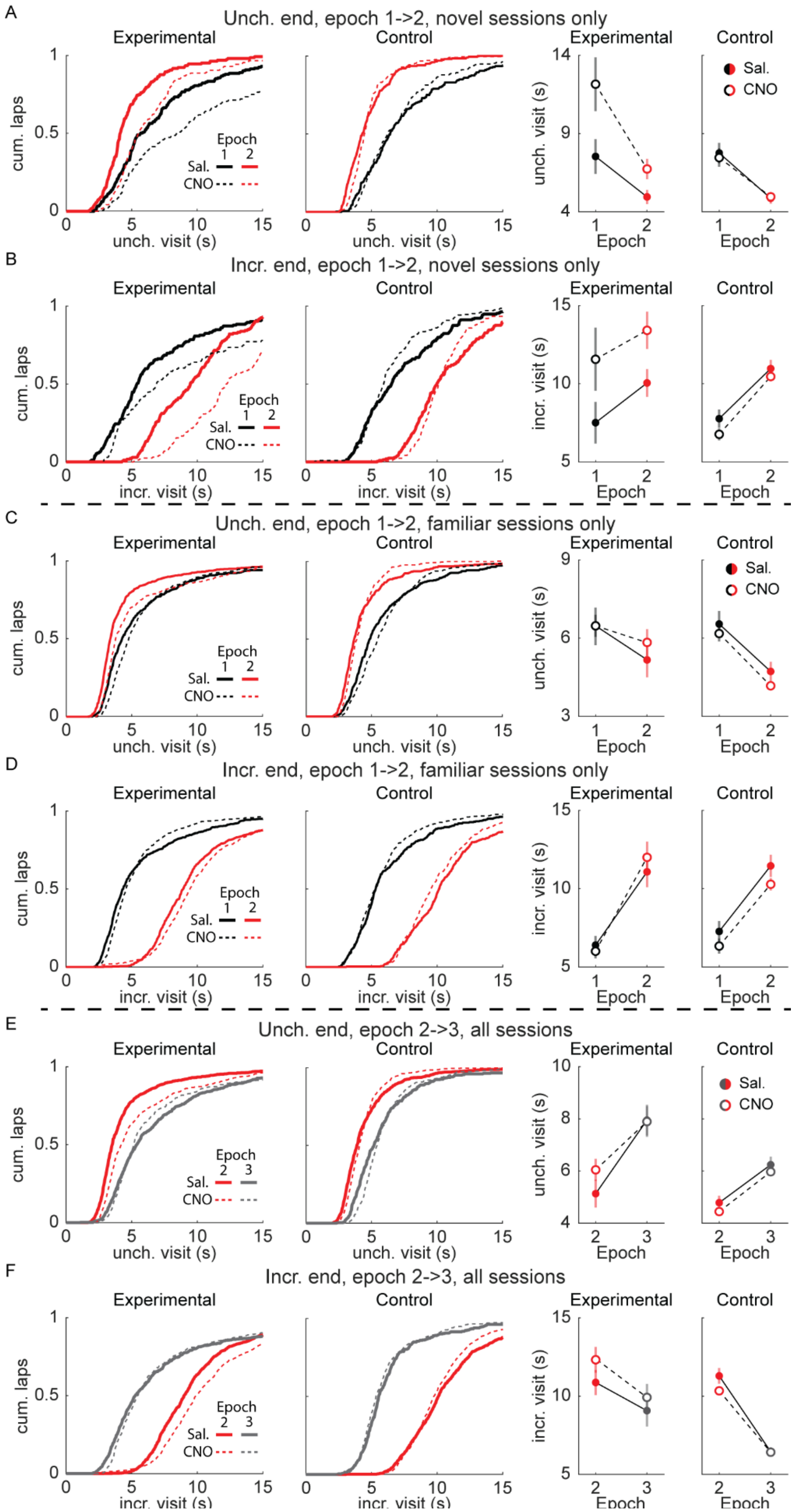


**Figure 1 – Supplement 2**

bioRxiv preprint doi: https://doi.org/10.1101/2024.06.04.597435; this version posted November 8, 2024. The copyright holder for this preprint (which was not certified by peer review) is the author/funder, who has granted bioRxiv a license to display the preprint in perpetuity. It is made available under aCC-BY 4.0 International license.

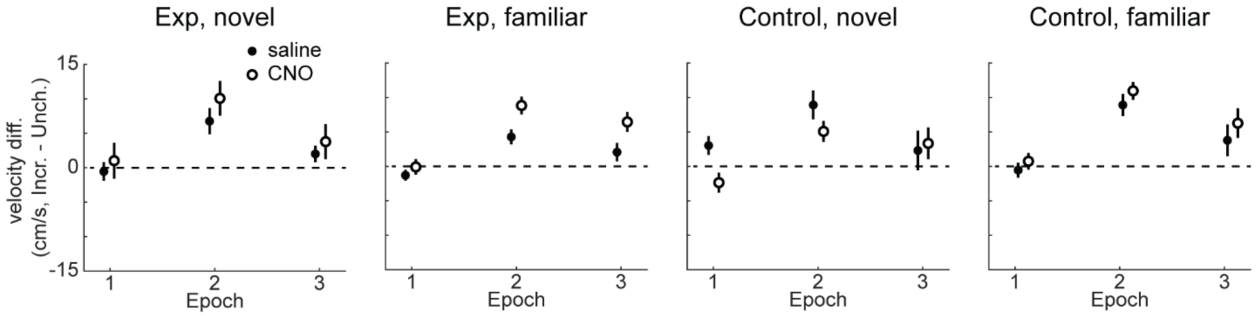


**Figure 1 – Supplement 3**

bioRxiv preprint doi: https://doi.org/10.1101/2024.06.04.597435; this version posted November 8, 2024. The copyright holder for this preprint (which was not certified by peer review) is the author/funder, who has granted bioRxiv a license to display the preprint in perpetuity. It is made available under aCC-BY 4.0 International license.

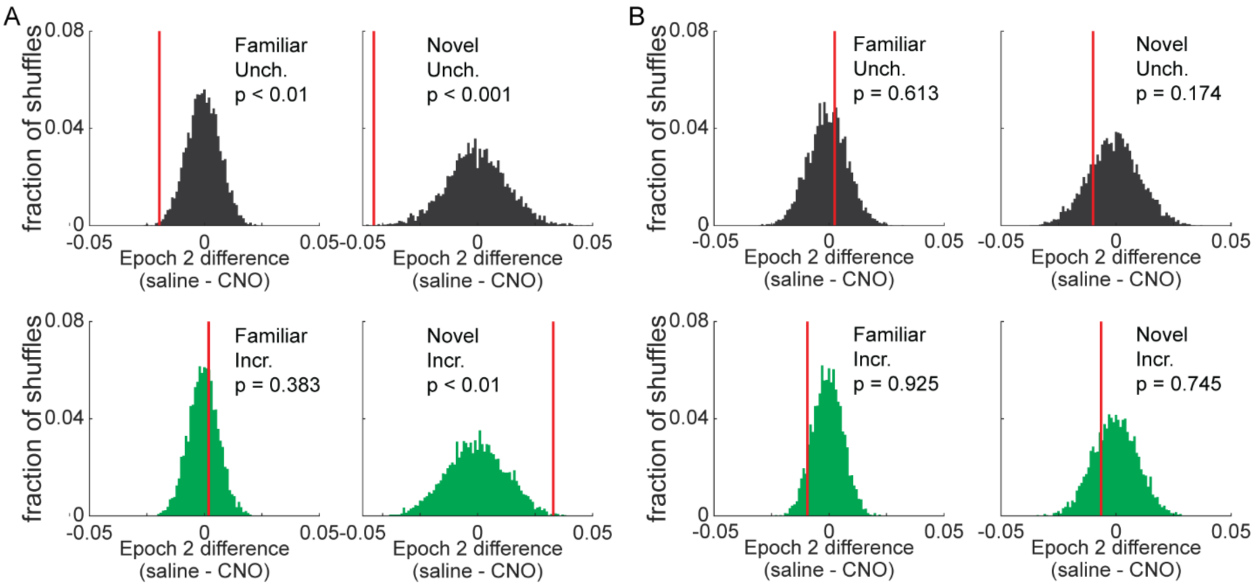


**Figure 2 – Supplement 1**

bioRxiv preprint doi: https://doi.org/10.1101/2024.06.04.597435; this version posted November 8, 2024. The copyright holder for this preprint (which was not certified by peer review) is the author/funder, who has granted bioRxiv a license to display the preprint in perpetuity. It is made available under aCC-BY 4.0 International license.

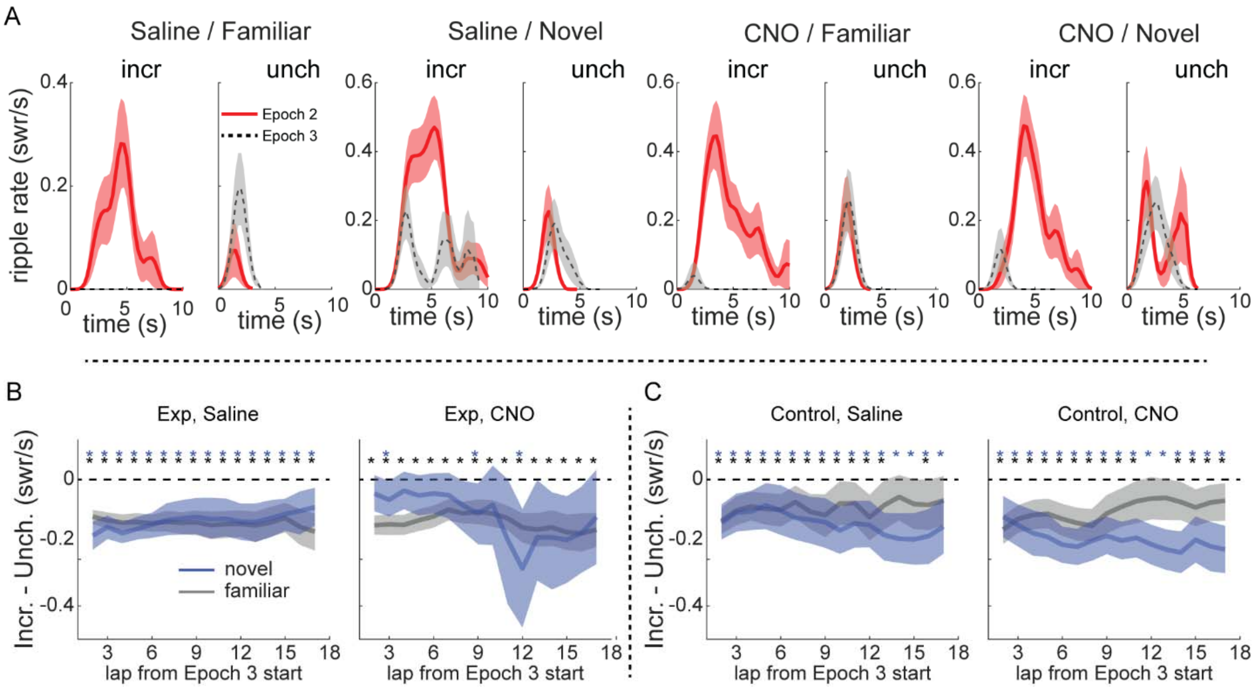


**Figure 2 – Supplement 2**

bioRxiv preprint doi: https://doi.org/10.1101/2024.06.04.597435; this version posted November 8, 2024. The copyright holder for this preprint (which was not certified by peer review) is the author/funder, who has granted bioRxiv a license to display the preprint in perpetuity. It is made available under aCC-BY 4.0 International license.

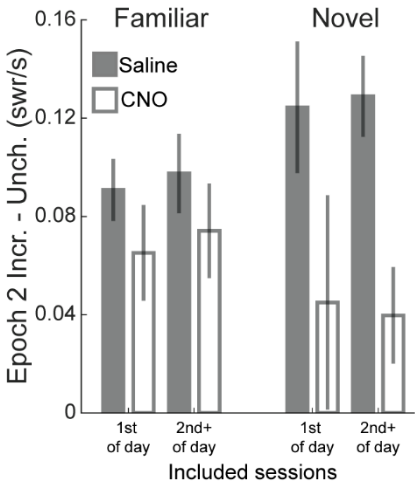


**Figure 2 – Supplement 3**

bioRxiv preprint doi: https://doi.org/10.1101/2024.06.04.597435; this version posted November 8, 2024. The copyright holder for this preprint (which was not certified by peer review) is the author/funder, who has granted bioRxiv a license to display the preprint in perpetuity. It is made available under aCC-BY 4.0 International license.

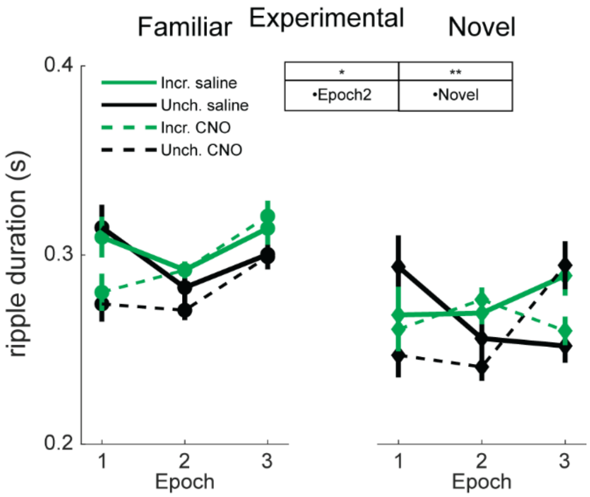


**Figure 2 – Supplement 4**

bioRxiv preprint doi: https://doi.org/10.1101/2024.06.04.597435; this version posted November 8, 2024. The copyright holder for this preprint (which was not certified by peer review) is the author/funder, who has granted bioRxiv a license to display the preprint in perpetuity. It is made available under aCC-BY 4.0 International license.

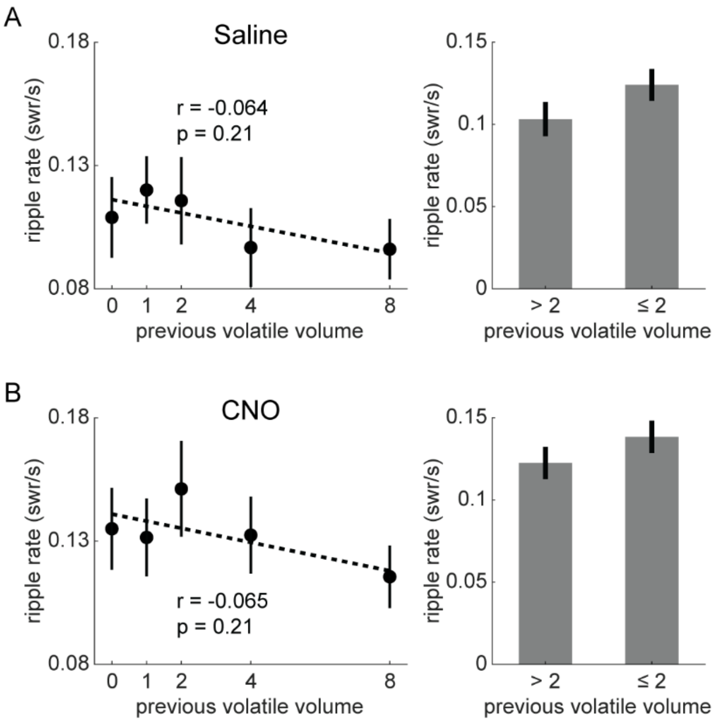


**Figure 3 – Supplement 1**

bioRxiv preprint doi: https://doi.org/10.1101/2024.06.04.597435; this version posted November 8, 2024. The copyright holder for this preprint (which was not certified by peer review) is the author/funder, who has granted bioRxiv a license to display the preprint in perpetuity. It is made available under aCC-BY 4.0 International license.

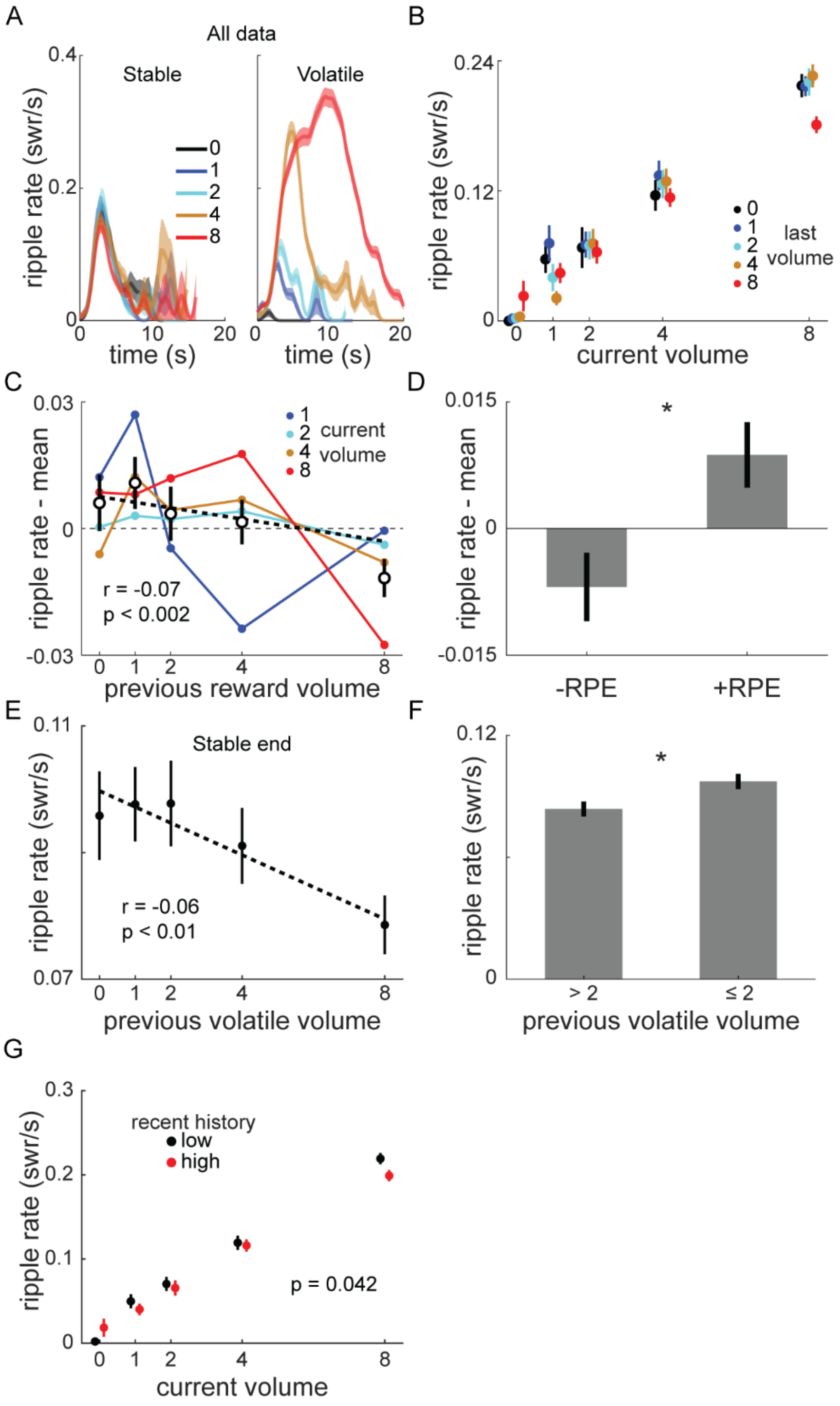


**Figure 3 – Supplement 2**

bioRxiv preprint doi: https://doi.org/10.1101/2024.06.04.597435; this version posted November 8, 2024. The copyright holder for this preprint (which was not certified by peer review) is the author/funder, who has granted bioRxiv a license to display the preprint in perpetuity. It is made available under aCC-BY 4.0 International license.

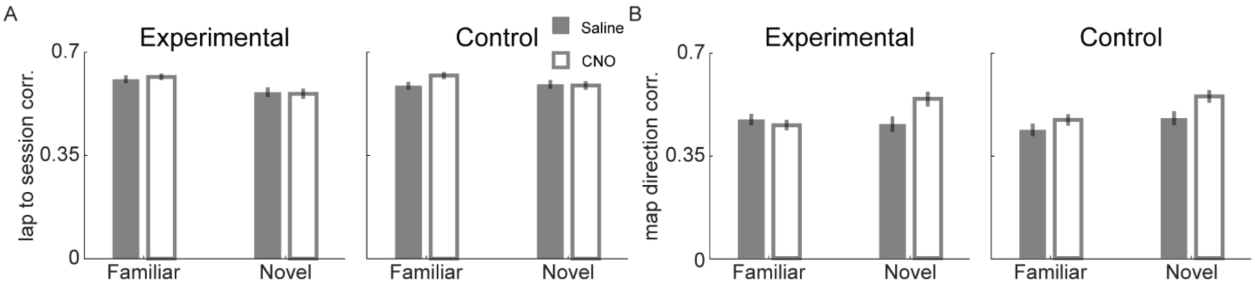


**Figure 4 – Supplement 1**

bioRxiv preprint doi: https://doi.org/10.1101/2024.06.04.597435; this version posted November 8, 2024. The copyright holder for this preprint (which was not certified by peer review) is the author/funder, who has granted bioRxiv a license to display the preprint in perpetuity. It is made available under aCC-BY 4.0 International license.

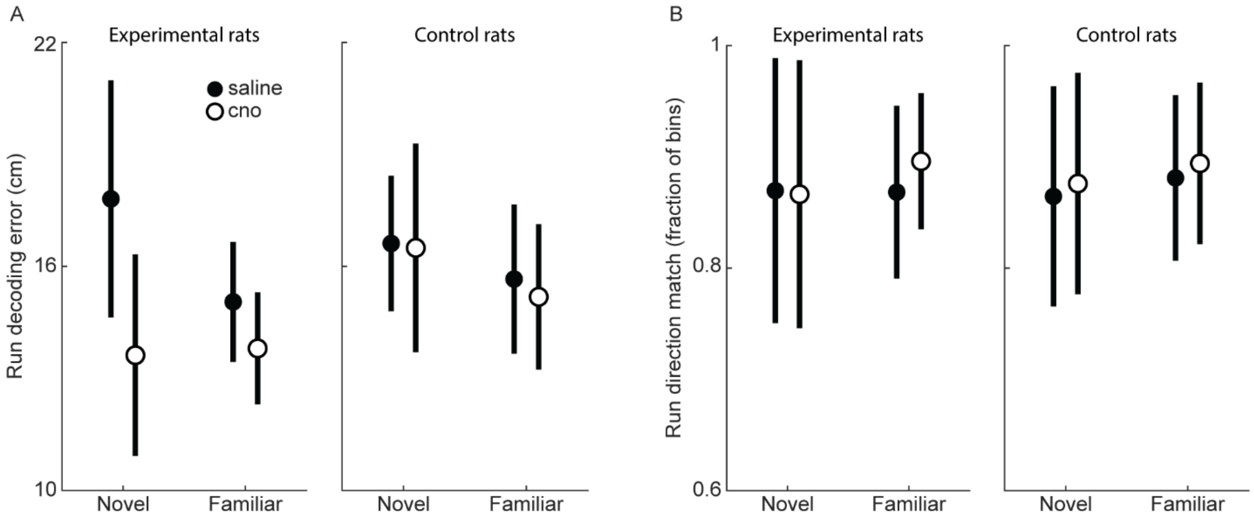


**Figure 4 – Supplement 2**

bioRxiv preprint doi: https://doi.org/10.1101/2024.06.04.597435; this version posted November 8, 2024. The copyright holder for this preprint (which was not certified by peer review) is the author/funder, who has granted bioRxiv a license to display the preprint in perpetuity. It is made available under aCC-BY 4.0 International license.

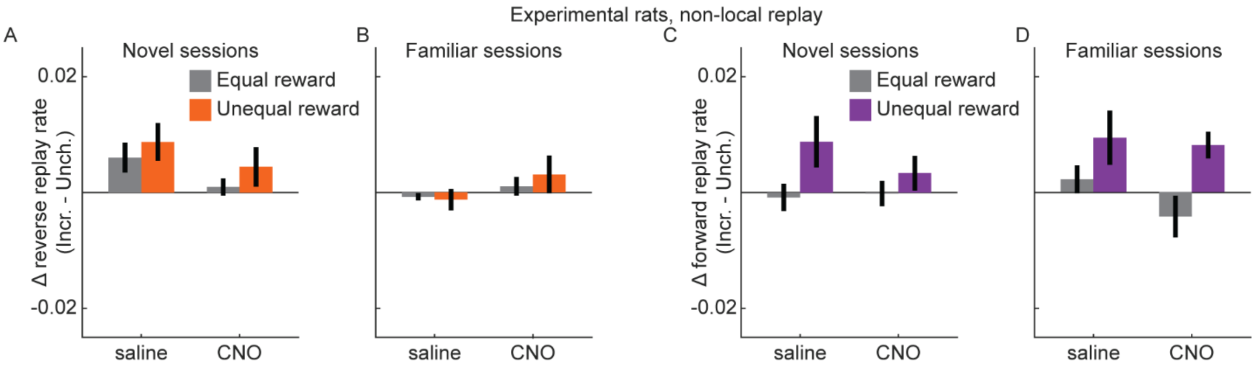


**Figure 4 – Supplement 3**
